# Temporal patterns of organ dysfunction after severe trauma

**DOI:** 10.1186/s13054-021-03586-6

**Published:** 2021-05-05

**Authors:** Jesper Eriksson, David Nelson, Anders Holst, Elisabeth Hellgren, Ola Friman, Anders Oldner

**Affiliations:** 1 Perioperative Medicine and Intensive Care, Karolinska University Hospital, Solna, 171 76 Stockholm, Sweden; 2 Section of Anaesthesiology and Intensive Care Medicine, Department of Physiology and Pharmacology, Karolinska Institute, Stockholm, Sweden; 3 KTH, Royal Institute of Technology, Stockholm, Sweden; 4 RISE, Research Institutes of Sweden, Gothenburg, Sweden

**Keywords:** Trauma, Critical care, Multiple organ dysfunction, Clustering, Data modelling

## Abstract

**Background:**

Understanding temporal patterns of organ dysfunction (OD) may aid early recognition of complications after trauma and assist timing and modality of treatment strategies. Our aim was to analyse and characterise temporal patterns of OD in intensive care unit-admitted trauma patients.

**Methods:**

We used group-based trajectory modelling to identify temporal trajectories of OD after trauma. Modelling was based on the joint development of all six subdomains comprising the sequential organ failure assessment score measured daily during the first two weeks post trauma. Further, the time for trajectories to stabilise and transition to final group assignments were evaluated.

**Results:**

Six-hundred and sixty patients were included in the final model. Median age was 40 years, and median ISS was 26 (IQR 17–38). We identified five distinct trajectories of OD. Group 1, mild OD (*n* = 300), median ISS of 20 (IQR 14–27), had an early resolution of OD and a low mortality. Group 2, moderate OD (*n* = 135), and group 3, severe OD (*n* = 87), were fairly similar in admission characteristics and initial OD but differed in subsequent OD trajectories, the latter experiencing an extended course and higher mortality. In group 3, 56% of the patients developed sepsis as compared with 19% in group 2. Group 4, extreme OD (*n* = 40), received most blood transfusions, had the highest proportion of shock at admission and a median ISS of 41 (IQR 29–50). They experienced significant and sustained OD affecting all organ systems and a 28-day mortality of 30%. Group 5, traumatic brain injury with OD (*n* = 98), had the highest mortality of 35% and the shortest time to death for non-survivors, median 3.5 (IQR 2.4–4.8) days. Groups 1 and 5 reached their final group assignment early, > 80% of the patients within 48 h. In contrast, groups 2 and 3 had a prolonged time to final group assignment.

**Conclusions:**

We identified five distinct trajectories of OD after severe trauma during the first two weeks post-trauma. Our findings underline the heterogeneous course after trauma and describe some potentially important clinical insights that are suggested by the groupings and temporal trajectories.

**Supplementary Information:**

The online version contains supplementary material available at 10.1186/s13054-021-03586-6.

## Background

Organ dysfunction (OD) is a major threat to critically injured patients surviving through the resuscitation phase. Organ dysfunction composes a significant clinical challenge in the intensive care unit (ICU)-treated trauma patient and is associated with prolonged length of stay (LOS), adverse outcomes, and demanding resource utilisation. With more patients surviving through the early trauma phase, due to advances in trauma care systems, OD becomes an increasing determinant of outcome. There is a known association between admission variables such as injury severity, shock, age and comorbidity, and subsequent OD [1–3]. However, the trajectories of OD, in terms of severity and composite temporal patterns and resolution, are complex and diverse. This aspect of trauma research has recently been identified as poorly investigated with a need for further studies [4].

Monitoring and treating in relation to temporal patterns of composite physiological states is inherent to ICU strategy. Each patient may be unique in their response. However, we expect human pathophysiology at some level to be groupable to distinct temporal patterns that are identifiable and reproducible. Understanding these patterns may help in timing of treatment strategies, pre-emptive identification of impending clinical decline [5–7], and to identify cohorts that in the future may require precision treatment approaches identified with predictive enrichment strategies. Despite these discernible prerequisites and notable pursuits, ICU and trauma research have to date been more focused on temporally static approaches to pattern recognition. As such, important steps towards identifying composite phenotypes of disease in the ICU have been taken for ICU-related diseases such as ARDS [8] and sepsis [9], but rely predominantly on early presentation data and patterns, and take less into account information content found in the temporal evolution of disease. Thus, there is a clear need to align pattern recognition with clinical temporal reasoning and human-interpretable models, even if this necessitates complex methods of time-series analysis and pattern recognition.

Group-based trajectory modelling (GBTM), as developed and described by Nagin and Odgers [10], generally fit polynomials, over time for a group of trajectories, using maximum likelihood estimation to a predefined number of categories. It is a specialised application of the finite mixture model, a probabilistic modelling to identify subpopulations. The optimal number of trajectories may to some extent be objectified using Akaike or Bayesian information criterion (AIC or BIC), with BIC driving more parsimonious solutions. It has been used for a number of real-world clinical data analyses to identify temporal trajectories [11–13] and affords a compelling method to reduce high-dimensional temporal data to a finite number of similar “type” trajectories.

The aim of this study was to analyse the temporal patterns of OD in ICU-treated critically injured patients. By using GBTM we attempt to identify and group sub-populations following a similar trajectory and characterise the temporal patterns of these groups in relation to both admission data and outcomes. In addition, we analyse the time from admission to trajectory group stabilisation in the identified sub-populations, as to discern if some subpopulations are less temporally predetermined and therefore potentially susceptible to treatment strategies during the ICU stay.

## Methods

### Setting

This retrospective cohort study of severely injured trauma patients was conducted at the ICU at the trauma centre at the Karolinska University Hospital, Stockholm, Sweden, using a prospectively collected dataset. This is the referral centre for severe trauma cases covering the largest urban region in Sweden with over two million inhabitants. The centre is equivalent to a Level-1 Trauma centre. Annually, approximately 1500 adult patients are admitted to the trauma centre. Of these patients, around 80% have indication for full trauma team activation, based on prespecified physiological parameters, anatomical injuries, and mechanism of injury. Approximately 300 admissions annually have an injury severity score (ISS) > 15.

### Study population and data collection

The study cohort consisted of trauma patients admitted to the ICU following initial resuscitation and, where indicated, interventional surgery. Patients 15 years or older with an expected ICU LOS of more than 24 h were included between February 2007 and November 2016. Data were retrieved during the ICU and, if applicable, subsequent high dependency unit (HDU) stay until discharge to general ward, death or up to 28 days after trauma, whichever occurred first. We excluded patients who were transferred to another hospital during their ICU or HDU stay since this resulted in loss of follow-up data.

All data were retrieved from the electronic patient data management system and prospectively entered into a database (TRAUMAREG) by research nurses. Data were then crosschecked and validated against the data sources (medical records) in retrospect by the researchers. If indicated, data were corrected in accordance with the source records. Trauma data such as ISS, mechanism of injury, admission blood pressure, admission Glasgow Coma Scale (GCS) were retrieved from the hospital trauma register. Data on comorbidity were retrieved from the patient charts.

### Definitions

Organ dysfunction was defined according to the sequential organ failure assessment (SOFA) score [14]. Comorbidity was defined as Charlson comorbidity index ≥ 1 as adapted by Gabbe et al. [15]. Injury severity was defined with ISS, using the abbreviated injury scale (AIS) version 2005. Infection was defined according to the international sepsis forum classification [16]. An infection was regarded as resolved when the patient no longer fulfilled the criteria according to this classification. Sepsis was defined according to the sepsis-3 definition [17] as an increase in SOFA score of two or more points in conjunction with an infection during the stay at the trauma ICU. Since trauma patients may have an inherently elevated SOFA score due to trauma, an increase in SOFA of two or more points from the previous day was used in the Sepsis-3 definition. If the patients SOFA score returned to the offset level, or if the patient no longer fulfilled the criteria for infection according to the international sepsis forum classification, the patient was no longer regarded as septic.

Massive transfusion was defined as ten or more units of packed red blood cells (PRBC) in the first 24 h. Shock on arrival was defined as systolic arterial blood pressure of less than 90 mm Hg on admission at the trauma unit. Trauma-induced coagulopathy (TIC) was defined as INR > 1.2 on admission. Surgery within 24 h was defined as surgical interventions involving a surgical team; thus, chest drain insertion and suturing of minor injuries were not included.

### Statistical methods

Group-based trajectory modelling was used to find unique trajectory groups of organ dysfunction after trauma, based on the daily values of the six organ subdomains in the SOFA score during the first 14 days after trauma. Thus, GBTM used the evolution, interrelationship, and temporal profiles across individual organ dysfunction trajectories in order to identify latent clusters following similar trajectories. Missing data on subdomains of SOFA were imputed by multiple imputation with chained equations using all available information of the other recorded subdomains of SOFA. When patients were discharged to the ward, we assigned 0 points in all six SOFA subdomains. For patients who died during the study time, data collected up until death were used in the model and no values were assumed or imputed after death.

Group-based trajectory modelling was performed with the package TRAJ for Stata (version 16.1). We fitted between one to eight trajectories using the censored normal distribution, yielding a probability for each patient belonging to a trajectory group, referred to as posterior probability of group membership (PPGM). Patients were assigned to the group corresponding to the highest PPGM. Modelling was based on fit statistics such as BIC, trajectory groups membership (no less then 5%), and average PPGM (no less than 70%).

The choice number of trajectories is in part inherently subjective, but importantly benefits from a clinically tractable number of trajectories and parsimonious solutions, without being simplified as to lose substantial information content. In our analysis BIC decreased steeply when increasing the number of trajectory groups up to four. Further, more than seven groups yielded groups below the five percent minimum group size. In addition, BIC decreased negligibly beyond five trajectory groups. Thus, the choice of five groups for the final model was partly based on substantive clinical interpretation of trajectory groups and a strive for parsimony as previously suggested by Nagin et al. [18]. For detailed methodological description please see Additional file 1.

In order to analyse the time for patients to find their specific trajectory of organ dysfunction, we investigated which day after admission the highest PPGM stabilised. We defined trajectory group assignment as stabilised when the highest PPGM did not change as compared to their final assignment.

Categorical data are presented as proportions and percentages. Continuous data are presented with median and interquartile ranges. Stata/MP 16.1 (Stata Corp, College Station, TX) was used for all analyses.

## Results

**Fig. 1:**
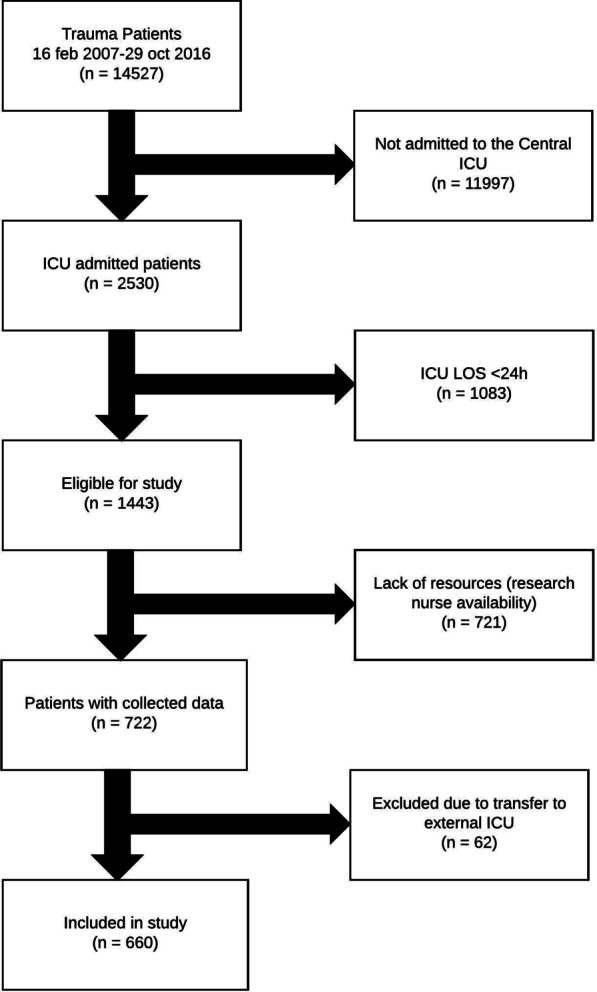
Flow chart. Flow chart of patients admitted to the intensive care unit (ICU) and included in the study

Six-hundred and sixty patients were included in the final model (see Fig. 1).

### Admission and demography data

Data on demography are depicted in Table 1. The median age was 40 years, and 22% had pre-existing comorbidity. Seventy-nine percent suffered blunt trauma with road traffic accidents as the most common mechanism of injury. The cohort had a male dominance and a high median ISS of 26. Fifteen percent were in shock on admission. One-fifth developed sepsis during the ICU stay. Median ICU LOS was 3.4 days, 28-day mortality 9.5%, and 1-year mortality 12.1%.

**Table 1:** Admission data

Factor	All patients	Group 1	Group 2	Group 3	Group 4	Group 5
*n*	660	300	135	87	40	98
Age	40 (27–56)	38 (26–51)	41 (27–56)	45 (28–63)	44 (31–60)	44 (27–64)
Sex (male)	517 (78%)	237 (79%)	102 (76%)	70 (80%)	35 (88%)	73 (74%)
Charlson comorbidity index ≥ 1	145 (22%)	59 (20%)	35 (26%)	24 (28%)	11 (28%)	16 (16%)
Injury mechanisms						
Traffic	273 (41%)	121 (40%)	58 (43%)	31 (36%)	19 (48%)	44 (45%)
Fall	113 (17%)	49 (16%)	19 (14%)	17 (19%)	6 (15%)	22 (22%)
Self-inflicted	109 (16%)	41 (14%)	22 (16%)	23 (26%)	8 (20%)	15 (15%)
Assault	83 (12%)	49 (16%)	14 (10%)	7 (8.0%)	3 (7.5%)	10 (10%)
Others	82 (12%)	40 (13%)	22 (16%)	9 (10%)	4 (10%)	7 (7.1%)
Intubated at scene	128 (19%)	31 (10%)	27 (20%)	12 (14%)	9 (22%)	49 (50%)
Blunt trauma	524 (79%)	234 (78%)	104 (77%)	73 (84%)	29 (72%)	84 (86%)
ISS, points	26 (17–38)	20 (14–27)	25 (18–38)	34 (22–43)	41 (29–50)	41 (29–54)
ISS > 15	545 (83%)	218 (73%)	115 (85%)	78 (90%)	39 (98%)	95 (97%)
AIS head ≥ 3	275 (42%)	70 (23%)	53 (39%)	47 (54%)	19 (48%)	86 (88%)
AIS chest ≥ 3	370 (56%)	139 (46%)	77 (57%)	55 (63%)	31 (78%)	68 (69%)
AIS abdomen ≥ 3	161 (24%)	70 (23%)	27 (20%)	27 (31%)	19 (48%)	18 (18%)
AIS spine ≥ 3	152 (23%)	52 (17%)	27 (20%)	34 (39%)	17 (42%)	22 (22%)
AIS lower extremity ≥ 3	207 (31%)	65 (22%)	54 (40%)	39 (45%)	24 (60%)	25 (26%)
Admission SAP, mmHg	123 (104–149)	130 (114–150)	120 (95–150)	126 (105–149)	90 (64–112)	120 (90–150)
Shock on arrival	99 (15%)	19 (6.3%)	26 (19%)	14 (16%)	18 (45%)	22 (22%)
Admission GCS	13 (8.0–15)	15 (12–15)	13 (8.0–15)	13 (8.0–15)	8.0 (3.0–14)	5.0 (3.0–8.0)
Admission creatinine, µM/L	92 (76–112)	90 (71–107)	87 (75–102)	101 (83–116)	119 (102–148)	92 (80–111)
Admission blood glucose, mM/L	8.8 (7.1–10)	8.2 (6.8–10)	8.9 (7.1–11)	9.1 (7.8–11)	9.8 (7.2–11)	10 (8.5–13)
Blood alcohol level > 0	171 (27%)	81 (28%)	31 (24%)	23 (28%)	11 (30%)	25 (26%)
Admission TIC	90 (15%)	32 (12%)	16 (14%)	18 (22%)	8 (24%)	16 (17%)
Admission INR	1.1 (1.0–1.2)	1.1 (1.0–1.2)	1.1 (1.0–1.2)	1.1 (1.0–1.2)	1.2 (1.1–1.2)	1.1 (1.0–1.2)
Admission platelet count, 10^9^/L	234 (188–282)	242 (199–288)	234 (190–283)	230 (190–276)	193 (157–271)	224 (179–268)
Admission fibrinogen level, g/L	2.2 (1.8–2.6)	2.2 (1.9–2.7)	2.2 (1.8–2.6)	2.2 (1.7–2.6)	1.5 (0.9–2.4)	1.9 (1.4–2.6)
Massive transfusion	109 (16%)	27 (9.0%)	23 (17%)	15 (17%)	27 (68%)	17 (17%)
Number of PRBC 24 h	2 (0–7)	0 (0–4)	2 (0–7)	4 (0–8)	12 (6–27)	2 (0–8)
Total fluid load 24 h, L	5.5 (3.5–8.6)	4.7 (2.7–7.2)	5.6 (33.7–8.7)	6.5 (4.2–9.5)	14 (8.1–21)	5.9 (4.0–9.0)
Surgery during the first 24 h	350 (53%)	140 (47%)	77 (57%)	56 (64%)	27 (68%)	50 (51%)

Admission data in the five groups. Continuous parameters presented as median (IQR), and categorical parameters presented as *n* (%). Admission refers to the admission to the trauma unit

ISS, injury severity score; AIS, abbreviated injury scale; SAP, systolic arterial blood pressure; shock on arrival defined as admission systolic blood pressure < 90 mmHg; GCS, Glasgow Coma Scale; TIC, trauma-induced coagulopathy; INR, international normalised ratio; PRBC, packed red blood cells

### Group assignments and characteristics

We identified five trajectories of OD after trauma based on unique combinations of the sub-trajectories of the six organ subdomains comprising the SOFA score. These five trajectory groups suggest distinct patterns of OD. Adding further trajectory groups was not deemed to add clinical information or resulted in too small group sizes, and less groups did not appear to capture the heterogeneity in the data. A comparison of models with one to eight groups is described in Additional file 1.

**Fig. 2:**
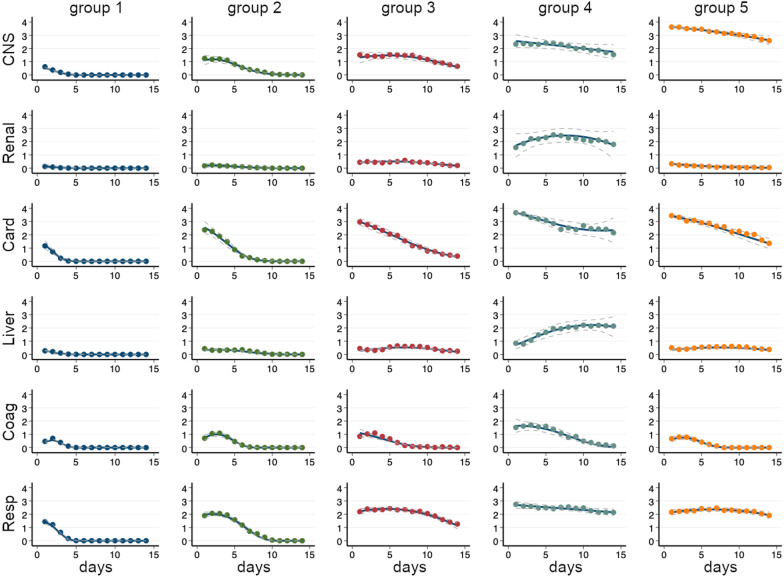
Trajectory group classification. The five identified trajectory groups of organ dysfunction represented by the columns. Sequential organ failure assessment (SOFA) points for each domain (*y*-axis) are shown for the first 14 days after trauma (*x*-axis). Final trajectory model (blue line) with corresponding 95% confidence intervals (dashed lines). Mean true observed SOFA score for each time point (dots). Central nervous system domain (CNS), renal domain (Renal), cardiovascular domain (Card), liver domain (Liver), coagulation domain (Coag), and respiratory domain (Resp). Reading example: Group 4 experienced relative stationary CNS SOFA scores during the first week. They experienced an increase in both renal and liver scores during the first week, after that renal scores gradually decreased, but liver scores continued to increase during the full study period of 14 days

**Fig. 3:**
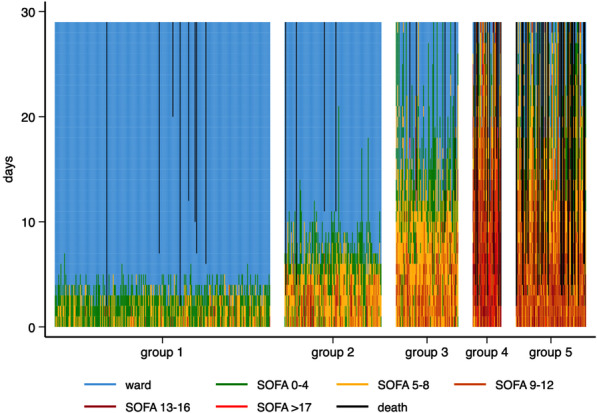
Heatmap of total sequential organ failure assessment score over time for the five identified groups. Each patient is represented by a single line coloured according to the legend, depicting sequential organ failure assessment (SOFA) score intervals, discharge to the ward or death. Days since trauma (*y*-axis), trajectory group (*x*-axis)

The final five-trajectory model displayed major differences between the five groups in admission and demographic characteristics (Table 1), temporal patterns of OD (Fig. 2), clinical course (Table 2), and outcomes (Table 3). According to the above findings the five groups were summarised as: group 1, mild OD; group 2, moderate OD; group 3, severe OD; group 4, extreme OD, and group 5, traumatic brain injury (TBI) with OD. The patterns over time for total SOFA scores over 28 days for individual patients in each group are depicted in a heat map (Fig. 3).

**Table 2:** Course in the intensive care and high dependency units

Factor	All patients	Group 1	Group 2	Group 3	Group 4	Group 5
N	660	300	135	87	40	98
APACHE II, points	15 (10–21)	12 (9–15)	14 (10–19)	16 (11–21)	23 (18–30)	24 (21–29)
Admission SOFA, points	6.0 (4.0–10)	4.0 (3.0–6.0)	7.0 (5.0–9.0)	8.0 (6.0–10)	12 (9.0–15)	11 (9.0–12)
Infection	284 (43%)	29 (9.7%)	79 (58%)	79 (91%)	34 (85%)	63 (64%)
Sepsis	139 (21%)	4 (1.3%)	25 (18%)	49 (56%)	29 (72%)	32 (33%)
Days with CNS SOFA ≥ 3	0.0 (0.0–3.0)	0.0 (0.0–0.0)	0.0 (0.0–2.0)	0.0 (0.0–4.0)	4.0 (1.0–9.0)	10 (4.0–17)
Days with renal SOFA ≥ 3	0.0 (0.0–0.0)	0.0 (0.0–0.0)	0.0 (0.0–0.0)	0.0 (0.0–0.0)	3.0 (1.0–14)	0.0 (0.0–0.0)
Days with cardiovascular SOFA ≥ 3	2.0 (0.0–5.0)	0.0 (0.0–0.0)	3.0 (0.0–5.0)	6.0 (3.0–8.0)	13 (6.5–16)	8.5 (3.0–13)
Days with liver SOFA ≥ 3	0.0 (0.0–0.0)	0.0 (0.0–0.0)	0.0 (0.0–0.0)	0.0 (0.0–0.0)	1.0 (0.0–7.0)	0.0 (0.0–0.0)
Days with coagulation SOFA ≥ 3	0.0 (0.0–0.0)	0.0 (0.0–0.0)	0.0 (0.0–0.0)	0.0 (0.0–0.0)	0.0 (0.0–2.0)	0.0 (0.0–0.0)
Days with respiratory SOFA ≥ 3	0.0 (0.0–3.0)	0.0 (0.0–0.0)	1.0 (0.0–2.0)	5.0 (2.0–8.0)	7.0 (3.5–12)	3.0 (1.0–8.0)
Proportion of days CNS SOFA ≥ 3	22%	7.3%	15%	14%	44%	78%
Proportion of days with renal SOFA ≥ 3	3.2%	0.3%	1.1%	3.4%	36%	1.5%
Proportion of days with cardiovascular SOFA ≥ 3	31%	15%	34%	36%	66%	60%
Proportion of days with liver SOFA ≥ 3	1.5%	0.0%	0.4%	1.4%	18%	1.1%
Proportion of days with coagulation SOFA ≥ 3	1.1%	0.5%	0.7%	0.4%	11%	0.0%
Proportion of days with respiratory SOFA ≥ 3	18%	4.4%	17%	32%	45%	35%
Days on vasopressor therapy	2.0 (0.0–5.0)	0.0 (0.0–0.0)	3.0 (0.0–5.0)	6.0 (4.0–8.0)	13 (6.5–16)	8.5 (3.0–13)
Days on mechanical ventilation	3.0 (1.0–8.0)	1.0 (0.0–2.0)	5.0 (3.0–6.0)	11.0 (8.0–14)	16.5 (10–22)	16 (5.0–23)
Days on CRRT	0.0 (0.0–0.0)	0.0 (0.0–0.0)	0.0 (0.0–0.0)	0.0 (0.0–0.0)	4.0 (0.0–18)	0.0 (0.0–0.0)

Data on the course in the intensive care unit (ICU) and if applicable high dependency unit (HDU) for the five groups. Continuous parameters presented as median (IQR), and categorical parameters presented as *n* (%)

APACHE, Acute Physiology and Chronic Health Evaluation; SOFA, sequential organ failure assessment; CNS, central nervous system; CRRT, continuous renal replacement therapy

**Table 3:** Outcomes

Factor	All patients	Group 1	Group 2	Group 3	Group 4	Group 5
*n*	660	300	135	87	40	98
ICU LOS	3.4 (1.9–8.0)	2.1 (1.5–2.9)	5.7 (4.6–7.7)	13 (9.9–16)	18 (7.3–27)	4.1 (1.9–16)
Hospital LOS	16 (9.4–30)	11 (6.9–17)	19 (13–26)	29 (21–42)	41 (16–72)	26 (3.9–46)
ICU mortality	47 (7.1%)	1 (0.3%)	1 (0.7%)	2 (2.3%)	10 (25%)	33 (34%)
28-day mortality	63 (9.5%)	8 (2.7%)	4 (3.0%)	4 (4.6%)	12 (30%)	35 (36%)
Time to death	4.3 (2.5–10)	6.6 (3.6–11)	7.6 (3.3–11)	12 (9.9–15)	7.4 (2.3–14)	3.5 (2.4–4.8)
1-year mortality	80 (12%)	13 (4.3%)	7 (5.2%)	6 (6.9%)	14 (35%)	40 (41%)

Data on outcomes for the five groups. Time to death refers to median time from trauma to death within 28 days for non-surviving patients. Continuous parameters presented as median (IQR), and categorical parameters presented as *n* (%)

ICU, intensive care unit; LOS, length of stay

### Group 1—mild organ dysfunction

This group with the lowest median age comprised 45% of the cohort and was characterised by a short period of organ dysfunction, despite a median ISS of 20 and 9% receiving massive transfusion. The need of organ supportive therapy and the incidence of sepsis were negligible. The outcomes were overall favourable with an ICU LOS of 2.1 days, 28-day and 1-year mortality of 2.7% and 4.3%, respectively.

### Group 2—moderate organ dysfunction

One-fifth of the patients was assigned to this group that largely resembled the characteristics of the overall cohort with a median age of 41, ISS 25, and 17% receiving massive transfusion. The temporal pattern was characterised by significant respiratory and cardiovascular dysfunction that was largely resolved within the first week. This was reflected in the need of organ supportive therapy with a median of three days on vasopressors and five days on mechanical ventilation. The sepsis incidence was 18.5%. Despite largely resembling the characteristics of the total cohort in terms of demography and admission data, the mortality was fairly low with a 28-day and 1-year mortality of 3.0 and 5.2%, respectively.

### Group 3—severe organ dysfunction

This group that comprised 13% of the cohort largely resembled the admission and demographic characteristics of group 2 but were older (median age 45) and had a higher median ISS [34]. The pattern of organ dysfunction was similar to group 2 during the initial phase but characterised by an extended course with an ICU LOS of 13 days and an incidence of sepsis of 56%. Despite high median age and severity of injury as compared with the overall cohort, the mortalities were lower with a 28-day and 1-year mortality of 4.6 and 6.9%, respectively.

### Group 4—extreme organ dysfunction

This small group of patients was characterised by severe injury and massive resuscitation. They received by far most transfusions and had the highest proportion of shock on arrival, lowest platelet, and fibrinogen levels as well as the highest incidence of TIC. The fluid load during resuscitation was significant. Their course was characterised by overwhelming and sustained organ dysfunction, including renal and liver dysfunction. The clinical course was highly complicated with a sepsis incidence of 72% and prolonged need of organ support including renal replacement therapy. The outcomes were poor with an ICU LOS of 18 days and an hospital LOS of over 40 days. The mortality rates were high with a 28-day and 1-year mortality of 30 and 35%, respectively. Despite the severity of injury and massive physiological derangement on admission, the median time to death for non-survivors was 7.4 days.

### Group 5—traumatic brain injury with organ dysfunction

Traumatic brain injury was the dominating injury in this group. Abbreviated injury scale head ≥ 3 was seen in 88%, and median admission GCS was 5. A fairly high proportion of chest injuries was also seen. Apart from protracted and severe central nervous system (CNS) dysfunction, cardiovascular and respiratory dysfunctions were common. The need of vasopressors and mechanical ventilation was significant. This group had the highest mortality rates, 36 and 41% at 28 days and 1 year, respectively. Time from admission to death for non-survivors was fairly short with a median of 3.5 days.

### Time to stabilisation and infectious complications

**Fig. 4:**
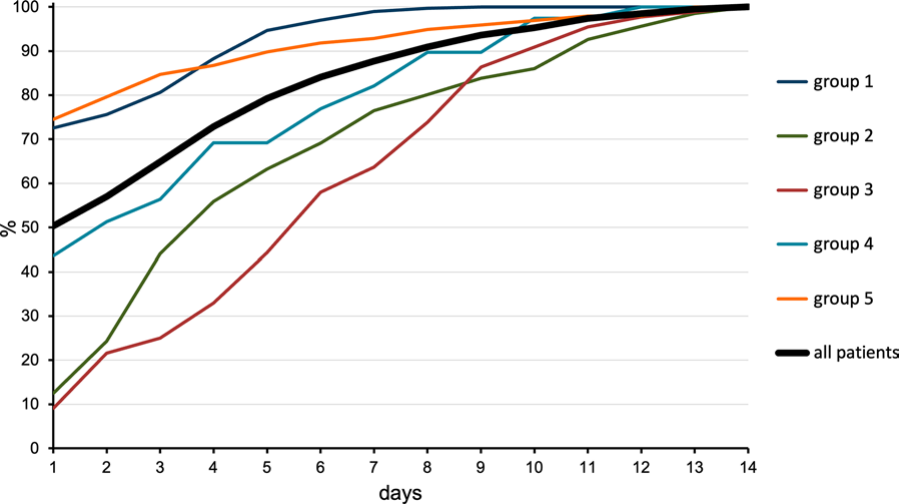
Trajectory stabilisation over time. Cumulative percentage of patients (*y*-axis) for whom the posterior probability of group membership stabilises at a given time point (*x*-axis). The legend depicts the colours for the respective trajectory groups as well as for the total cohort

The proportion of patients being assigned to their trajectory group over time (Fig. 4) showed major differences between groups. Groups 1 and 5, the groups with the lowest and highest mortalities, found their trajectory at an early stage. For groups 2 and 3, moderate and severe organ dysfunction, stabilisation into the final trajectory groups was seen at a much later stage. Less than half of the patients were assigned to their final trajectory by day three. Onset of infections and sepsis typically occurred a few days after admission, and the incidence varied notably between these two groups (see heatmap, Additional file 2).

## Discussion

This is to our knowledge the first study using temporal clustering based on specific organ systems after severe trauma. We identified five groups with contrasting organ dysfunction trajectories, admission characteristics, and outcomes. The time to stabilisation into the final trajectory group differed notably between groups. This reflects the complexity of predicting the clinical course for some subsets of critically injured patients, but also provide opportunities to identify subgroups that may have avoidable deterioration or could benefit from specific treatments.

Post-injury OD is a well-recognised complication following severe trauma. This challenge is expected to increase with improved initial survival in modern trauma care systems, an ageing population, and rising burdens of comorbidity. The aetiology is not fully elucidated. There is a well-known association between injury severity, shock, and red blood cell transfusion [19, 20]. A factor advocated as a trigger of organ dysfunction is damage-associated molecular patterns (DAMPs) [21]. Substances like mitochondrial DNA, histones, and HMGB1 normally contained in the intra-cellular compartment released during tissue injury and hypoperfusion triggering an inflammatory response evoked by the innate immune system [22, 23]. There is an association between levels of circulating DAMPs and subsequent OD following trauma [22]. In addition, events occurring after trauma, such as infections and sepsis, may contribute to further OD and complicate prognostication of the clinical course.

Previous studies on post-injury OD are diverse. The use of different scoring systems in the assessment of OD complicates comparison between studies [24, 25]. There is also controversy as to the current trending of OD incidence, where some studies report that OD is a declining problem [26] and others report the contrary [27, 28]. In aggregate, in critically ill trauma patients a substantial unexplained heterogeneity of clinical course has been identified and advocated as a specific research challenge, calling for more exhaustive analyses [4, 29].

Few studies have addressed the pattern of OD over time, apart from onset times and impact on outcomes. Two previous studies analysing temporal patterns of OD did not assess specific organ systems but used scores of total OD [30, 31]. Thus, comparing the current study to previous work is problematic. Some resemblances are, however, noteworthy. One study of 440 UK patients, based on hierarchal clustering of total SOFA scores, identified three groups with contrasting trajectories, one dominated by TBI patients, one with severe injury and subsequently protracted OD and poor outcomes, and one with less injury, OD, and better outcomes [30]. In the current study we wanted to analyse both the temporal and organ-specific patterns of OD in an objective and discerning manner. The GBTM method has been shown to compare well with other methods of longitudinal models [32]. We used GBTM based on SOFA score for individual organs over time in order to identify groups with similar patterns. This method enabled us to use the full range of the temporal data by unsupervised differentiation of patients into latent trajectories, based on the joint inter-related development of their organ-based outcome patterns. Thus, this method allows to identify group assignment based on similarities in temporal combinations of specific patterns for each organ system, as opposed to previous studies using total OD without assessing specific organ systems. However, clustering of heterogeneous individual trajectories into fewer summarising trajectories is a task of dimensionality reduction and will always require some extent of simplification and approximation. With the GBTM method these trajectories were not assumed a priori as in subjective, often expert-based, categorisation of patients, but based on the true temporal profiles of the patients. Additionally, the method provides a measure of individual and group probability and, thus, certainty of trajectory group assignment.

This method generated five groups with different temporal patterns and outcomes. Group 1, the mild OD group had a favourable outcome with early resolution of OD and low mortality. Within this group there were, however, patients with diverging admission characteristics with some patients having an ISS of 50 and some being transfused with > 20 PRBCs units during the first 24 h. This cohort had the lowest median age and found their group trajectory rapidly after admission, suggesting that this subset of trauma patients can be identified at an early stage. Group 2 and group 3, the moderate and severe OD groups, were fairly similar in their admission parameters and had by far the longest time to trajectory stabilisation. These patients were in the mid-range in terms of injury severity and initial OD load as compared with the total cohort. However, the incidence of sepsis differed notably between the two groups, occurring more often in the severe OD group. The onset typically occurred a few days after admission possibly influencing trajectories and delaying stabilisation. These differences in time seen for trajectories to stabilise appear to reflect the heterogeneity amongst severely injured trauma patients that is often recognised clinically, where some patients develop an unexpectedly complicated clinical course, whereas others, with similar admission characteristics, do not. Our study suggests that while certain subsets of trauma patients will enter a clinical course that may be fairly well predicted in the early phase (group 5, TBI with OD, and group 1, mild OD), others have an undecided course (group 2, moderate OD, and group 3, severe OD) that may potentially be positively or negatively influenced by post-resuscitation events or treatments. This could suggest a potential window of therapy in these subgroups. In our study, sepsis appears related to a higher OD severity in the late stabilisers, which leads us to hypothesise that infectious complications may be a driver. The important implication of this is that potentially avoidable complications, arising after ICU admission, could be a reason that a significant number of patients with seemingly similar initial trajectories experienced different later patterns of organ failure. Consequently, their trajectories will be harder to define at an early stage.

The group of highly injured patients with extreme OD are readily recognisable clinically and pose a challenging entity in the ICU. In this study, group 4, extreme OD, was small and characterised by high injury severity where chest injuries were seen in 77%. Almost half of the patients were in shock at admission and two-thirds received massive transfusion, and these figures are in striking contrast to all other groups. The subsequent OD was considerable and sustained, affecting all organ systems. The incidence of liver and renal dysfunction was significant and increased during several days after admission. This was not seen in any other groups. Arguably, the injury severity, dose of shock, and massive transfusion seen in this group would be expected to generate a considerable release of DAMPs possibly contributing to the prominent OD seen in the post-injury phase. As expected, this group had a high mortality, but the median time to death was over a week for non-survivors, indicating a complicated clinical course, as seen in the vast need of organ supportive therapy. This small group of patients required a massive use of ICU recourses. The implication of such a highly identifiable group that distinguishes itself fairly early, and where mortality is extremely high, is that inflammatory and coagulopathic effects of shock and massive transfusion may need also to be a highlighted focus area of research in future trauma patients.

Traumatic brain injury represents a specific group of ICU patients. In this study, group 5, TBI with OD, had the worst outcomes of all groups in terms of mortality. Apart from CNS dysfunction subsequent to significant head injury the load of cardiovascular and respiratory OD was significant. This could in part be a confounder explained by the use of vasopressors to titrate optimal cerebral perfusion pressure and the need of extended controlled ventilation, as well as an expected high rate of respiratory infections, inflating non-CNS organ failure scores [33]. Traumatic brain injury is reported to be the main cause of death after trauma, and a high mortality in this group is expected [34, 35]. The median time to death was fairly short with 3.5 days for non-survivors as compared to group 4, suggesting a considerable proportion of refractory severe TBI. This group of patients with an almost 90% incidence of AIS head > 3 found their trajectory rapidly, and over 80% of these patients were assigned to their trajectory group by day 2. Thus, the TBI cohort is, not unexpectedly, highly discernible and exhibits an early defined trajectory of SOFA organ scores.

The methodology used here to group temporal trajectories provides a means with which to identify and separate phenotypes of trauma-related OD. Our findings exemplify the possibility of using not only expert-based or baseline data for clustering of patients, but instead to utilise the full range of available temporal data. Future research should be focused on external validation of our findings and using temporal clustering in other heterogeneous groups of intensive care patients such as for example cardiac arrest, sepsis, or ARDS.

### Strengths

We used a high-resolution database where all data were retrospectively validated by the researchers. Missing data were limited. There was no loss to follow-up in our patient cohort after excluding those patients transferred to other ICUs. All data were collected prospectively. The use of GTBM utilises all available organ score data up to 14 days and minimises subjective or eminence-based identification of organ patterns.

### Limitations

This study has several limitations. This is a single-centre study of trauma patients admitted to the ICU which limits generalisability. The TRAUMAREG database was run as a project and closed in November 2016, and we could not include all eligible patients due to shortage of research staff, which might have introduced a selection bias. Further, we cannot exclude that changes occurred in the trauma system and ICU care during the time period data were collected. We did not have any data after discharge to the general wards and our choice to assign zero SOFA points when patients were discharged to the wards could be debated. We chose to use the sub-domains of the SOFA score for building our model. SOFA score, however, commonly used in critical care and validated in trauma patients, is based on predefined weighted levels. Using more organ descriptive data with continuous parameters, might have revealed other patterns of organ dysfunction. We did not have information on more detailed outcomes, such as function scores, limiting the outcome analyses. Our definition of resolved infection could be debated, and it is possible that patients were infected for longer periods than we have accounted for. Further, we had no data on baseline anti-coagulative medication possibly influencing admission INR values. Due to increasing model complexity, we could not model longer periods than 14 days, and it is possible that the distribution between trajectory groups would have been different if this would have been possible. Finally, the number of trajectory groups is, at least to a degree, based on subjective judgement. It is, however, important to bear in mind that the goal of GBTM is not to identify the "true" number of groups in a sample, which may not exist, but rather to cluster patients following approximately the same trajectory.

The dataset used in the study is not immediately publicly accessible (see declarations below).

## Conclusions

We performed longitudinal clustering of severely injured trauma patients using data from all affected organs. Our study strives to objectify the temporal patterns of OD after admission to the ICU after severe trauma. Five distinct trajectories of organ failure after trauma were identified. Notable differences in admission characteristics, clinical course, and outcomes between the five groups generated by the GBTM modelling were seen. We found and describe some potentially important insights that were suggested by the groupings and temporal trajectories. These findings indicate subsets of patients with an initial undefined clinical course that might benefit from targeted support and future research focus. The findings also underline the heterogeneous course after trauma and the challenges faced in prognosticating the clinical course of these patients.

## Supplementary Information

**Additional file 1.** Methods supplement. Figures and fit statistics of 1–8 trajectory group models. GRoLTS checklist.

**Additional file 2.** Heatmap depicting infectious complications. Each patient is represented by a single line coloured according to the legend, depicting infectious complications, discharge to ward, or death. White lines depict missing data for infection. Days since trauma (*y*-axis), trajectory group (*x*-axis).

## Data Availability

The statistical analysis and code syntax used are available from the authors upon reasonable request. The data are not publicly available due to ethical restrictions and legal constraints. Readers may contact Dr Eriksson for reasonable requests for the data. De-identified data may be provided after approval from the ethical review board.

## References

[1] Frohlich M, Lefering R, Probst C, Paffrath T, Schneider MM, Maegele M (2014). Epidemiology and risk factors of multiple-organ failure after multiple trauma: an analysis of 31,154 patients from the TraumaRegister DGU. J Trauma Acute Care Surg.

[2] Vogel JA, Liao MM, Hopkins E, Seleno N, Byyny RL, Moore EE (2014). Prediction of postinjury multiple-organ failure in the emergency department: development of the Denver Emergency Department Trauma Organ Failure score. J Trauma Acute Care Surg.

[3] Brattstrom O, Granath F, Rossi P, Oldner A (2010). Early predictors of morbidity and mortality in trauma patients treated in the intensive care unit. Acta Anaesthesiol Scand.

[4] Asehnoune K, Balogh Z, Citerio G, Cap A, Billiar T, Stocchetti N (2017). The research agenda for trauma critical care. Intensive Care Med.

[5] Liu R, Greenstein JL, Granite SJ, Fackler JC, Bembea MM, Sarma SV (2019). Data-driven discovery of a novel sepsis pre-shock state predicts impending septic shock in the ICU. Sci Rep.

[6] Meyer A, Zverinski D, Pfahringer B, Kempfert J, Kuehne T, Sündermann SH (2018). Machine learning for real-time prediction of complications in critical care: a retrospective study. Lancet Respir Med.

[7] Komorowski M, Celi LA, Badawi O, Gordon AC, Faisal AA (2018). The Artificial Intelligence Clinician learns optimal treatment strategies for sepsis in intensive care. Nat Med.

[8] Calfee CS, Delucchi K, Parsons PE, Thompson BT, Ware LB, Matthay MA (2014). Subphenotypes in acute respiratory distress syndrome: latent class analysis of data from two randomised controlled trials. Lancet Respir Med.

[9] Seymour CW, Kennedy JN, Wang S, Chang CH, Elliott CF, Xu Z (2019). Derivation, validation, and potential treatment implications of novel clinical phenotypes for sepsis. JAMA.

[10] Nagin DS, Odgers CL (2010). Group-based trajectory modeling in clinical research. Annu Rev Clin Psychol.

[11] Jha RM, Elmer J, Zusman BE, Desai S, Puccio AM, Okonkwo DO (2018). Intracranial pressure trajectories: a novel approach to informing severe traumatic brain injury phenotypes. Crit Care Med.

[12] Rod NH, Bengtsson J, Budtz-Jørgensen E, Clipet-Jensen C, Taylor-Robinson D, Andersen AN (2020). Trajectories of childhood adversity and mortality in early adulthood: a population-based cohort study. Lancet.

[13] Tchalla AE, Dufour AB, Travison TG, Habtemariam D, Iloputaife I, Manor B (2014). Patterns, predictors, and outcomes of falls trajectories in older adults: the MOBILIZE Boston Study with 5 years of follow-up. PLoS ONE.

[14] Vincent JL, Moreno R, Takala J, Willatts S, De Mendonca A, Bruining H (1996). The SOFA (Sepsis-related Organ Failure Assessment) score to describe organ dysfunction/failure. On behalf of the Working Group on Sepsis-Related Problems of the European Society of Intensive Care Medicine. Intensive Care Med..

[15] Gabbe BJ, Magtengaard K, Hannaford AP, Cameron PA (2005). Is the Charlson Comorbidity Index useful for predicting trauma outcomes?. Acad Emerg Med Off J Soc Acad Emerg Med.

[16] Calandra T, Cohen J (2005). International Sepsis Forum Definition of Infection in the ICUCC. The international sepsis forum consensus conference on definitions of infection in the intensive care unit. Crit Care Med.

[17] Singer M, Deutschman CS, Seymour CW, Shankar-Hari M, Annane D, Bauer M (2016). The third international consensus definitions for sepsis and septic shock (Sepsis-3). JAMA.

[18] Nagin DS, Jones BL, Passos VL, Tremblay RE (2018). Group-based multi-trajectory modeling. Stat Methods Med Res.

[19] Eguia E, Cobb AN, Baker MS, Joyce C, Gilbert E, Gonzalez R (2019). Risk factors for infection and evaluation of Sepsis-3 in patients with trauma. Am J Surg.

[20] Patel SV, Kidane B, Klingel M, Parry N (2014). Risks associated with red blood cell transfusion in the trauma population, a meta-analysis. Injury.

[21] Vourc'h M, Roquilly A, Asehnoune K (2018). Trauma-induced damage-associated molecular patterns-mediated remote organ injury and immunosuppression in the acutely ill patient. Front Immunol.

[22] Simmons JD, Lee YL, Mulekar S, Kuck JL, Brevard SB, Gonzalez RP (2013). Elevated levels of plasma mitochondrial DNA DAMPs are linked to clinical outcome in severely injured human subjects. Ann Surg..

[23] Sharma SK, Naidu G (2016). The role of danger-associated molecular patterns (DAMPs) in trauma and infections. J Thorac Dis.

[24] Frohlich M, Wafaisade A, Mansuri A, Koenen P, Probst C, Maegele M (2016). Which score should be used for posttraumatic multiple organ failure?—Comparison of the MODS, Denver- and SOFA- Scores. Scand J Trauma Resusc Emerg Med.

[25] Hutchings L, Watkinson P, Young JD, Willett K (2017). Defining multiple organ failure after major trauma: a comparison of the Denver, Sequential Organ Failure Assessment, and Marshall scoring systems. J Trauma Acute Care Surg.

[26] Dewar DC, Tarrant SM, King KL, Balogh ZJ (2013). Changes in the epidemiology and prediction of multiple-organ failure after injury. J Trauma Acute Care Surg.

[27] Oyeniyi BT, Fox EE, Scerbo M, Tomasek JS, Wade CE, Holcomb JB (2017). Trends in 1029 trauma deaths at a level 1 trauma center: impact of a bleeding control bundle of care. Injury.

[28] Di Saverio S, Gambale G, Coccolini F, Catena F, Giorgini E, Ansaloni L (2014). Changes in the outcomes of severe trauma patients from 15-year experience in a Western European trauma ICU of Emilia Romagna region (1996–2010). A population cross-sectional survey study. Langenbecks Arch Surg.

[29] Deutschman CS, Ahrens T, Cairns CB, Sessler CN, Parsons PE (2012). Multisociety task force for critical care research: key issues and recommendations. Chest.

[30] Cole E, Gillespie S, Vulliamy P, Brohi K (2020). Multiple organ dysfunction after trauma. Br J Surg.

[31] Liu D, Namas RA, Vodovotz Y, Peitzman AB, Simmons RL, Yuan H (2020). Unsupervised clustering analysis based on MODS severity identifies four distinct organ dysfunction patterns in severely injured blunt trauma patients. Front Med.

[32] Elmer J, Jones BL, Nagin DS (2020). Comparison of parametric and nonparametric methods for outcome prediction using longitudinal data after cardiac arrest. Resuscitation.

[33] Hyllienmark P, Brattström O, Larsson E, Martling CR, Petersson J, Oldner A (2013). High incidence of post-injury pneumonia in intensive care-treated trauma patients. Acta Anaesthesiol Scand.

[34] Lansink KW, Gunning AC, Leenen LP (2013). Cause of death and time of death distribution of trauma patients in a level I trauma centre in the Netherlands. Eur J Trauma Emerg Surg.

[35] Dutton RP, Stansbury LG, Leone S, Kramer E, Hess JR, Scalea TM (2010). Trauma mortality in mature trauma systems: are we doing better? An analysis of trauma mortality patterns, 1997–2008. J Trauma.

[36] von Elm E, Altman DG, Egger M, Pocock SJ, Gotzsche PC, Vandenbroucke JP (2007). The Strengthening the Reporting of Observational Studies in Epidemiology (STROBE) statement: guidelines for reporting observational studies. Lancet.

[37] van de Schoot R, Sijbrandij M, Winter SD, Depaoli S, Vermunt JK (2017). The GRoLTS-Checklist: guidelines for reporting on latent trajectory studies. Struct Equ Modeling.

