# Supplementary material for: Temporal patterns of organ dysfunction after severe trauma
Source: Crit Care. 2021 May 5;25:165. doi: 10.1186/s13054-021-03586-6 (PMC8101241; doi:10.1186/s13054-021-03586-6)
Supplement: Supplementary file 1 — Additional file 1. Methods supplement. Figures and fit statistics of 1–8 trajectory group models. GRoLTS checklist. [file 13054_2021_3586_MOESM1_ESM.docx]

**Methods, supplement**

*Missing data*

Data on SOFA, used in model construction, was found missing at between 0-9%, depending on the variable (supplement table 1 in this document). We found no indication that missingness was other than at random. In support of this, missingness was not significantly associated with mortality at 28 days post trauma in logistic regression, with the dependent covariates, subdomains of SOFA set to 0 or 1 depending on if the variable was missing or not. Prior to clustering we therefore assumed that intermittently missing data during the ICU stay were conditional on observed variables and as such "missing at random". These intermittently missing data of subdomains of SOFA, during the ICU or HDU stay, were imputed by multiple imputation with chained equations (MICE) using all available information of the other recorded subdomains of SOFA. This was performed 10 times creating 10 complete datasets. The mean of the imputed datasets was used in model construction.

|  | **Missing n (%) / Total** |
| --- | --- |
| SOFA CNS | 0 (0%) / 6175 |
| SOFA Renal | 211 (3.4%) / 6175 |
| SOFA Cardiovascular | 2 (0%) / 6175 |
| SOFA Liver | 567 (9.2%) / 6175 |
| SOFA Coagulation | 214 (3.5%) / 6175 |
| SOFA Respiratory | 3 (0%) / 6175 |

Supplementary table 1. Missing values used in model building

*Temporal analysis*

After careful consideration and weighting against other methods, including PCA and k-mean clustering, we chose to use Group-Based Trajectory Modelling (GBTM) when modelling. This technique has attracted growing interest since its introduction by Nagin in 1993. We initially explored Principal Component Analysis using Stata v 16.1 and longitudinal K-means using the *kml* package in R (R Foundation for Statistical Computing, Vienna). Research using GBTM has been shown to perform well against other techniques of longitudinal modelling (1).

GBTM is a subset of finite mixture models. Another commonly used finite mixture model is growth mixture modelling, GMM. In contrast to GMMs, GBTMs do not allow for random effects. Additionally, they assume equal variance in the groups and over time. This might appear a disadvantage. However, one drawback with GMM is that estimation is more complex, involves more parameters and is more likely to experience convergence issues.

Since we primarily were interested in differences between trajectories (i.e., classes) of organ failure, and not differences within-classes, the GBTM offered distinct advantages. GBTM is also recommended in the presence of more complex models that fail to converge. We experienced convergence issues since we wanted to incorporate all six sub-domains of SOFA in our model, and at the same time model longer periods of time.

Therefore, GBTM was chosen, and the models were restricted to 14 days post trauma. For a non-technical summary and comparison between common techniques of trajectory modelling, please refer to *Group based modelling in clinical research* by Nagin and Odgers. (2)

*Group-based trajectory modelling*

GBTM aims to identify groups of individuals with similar, inter-related, temporal trajectories of one (single-trajectory model) or many (multi-trajectory model) measurements over time. The assumption is that there exist markedly distinct latent trajectory groups within the population of interest. Maximum likelihood is used for model parameter estimation.

Trajectory groups are latent strata. Individuals do not *belong* to specific trajectory groups, rather they are assigned a probability of group membership; Posterior probability of group membership (PPGM). After model building, patients were thus assigned to the trajectory group for which they had the highest probability of membership. For a more complete background on GBTM including the mathematical foundation please refer to Nagin et al (3).

*Model building*

Patients did not contribute additional data to the models after dropout (i.e., death) and were pragmatically assigned as missing thus not further affecting class probabilities. However, when patients recovered and were admitted to the regular ward, we assumed zero points in SOFA for all six organs for the rest of the study period, pragmatically assuming a full recovery. Assigning maximum points to all organs after death resulted in that all patients who died ended up in the same trajectory group, regardless of time to death or organ dysfunction pattern before death.

In the initial process of model building, we estimated single-trajectory models with varying number of trajectory groups for each of the six SOFA-domains separately and for the first 14 days after trauma. Using more days in the model resulted in increasing model complexity and convergence issues. This first step was done in order to visualize and understand the variability in the data and thus appreciate the minimum number of trajectory groups needed to be represented for each organ domain in the multi-trajectory model.

Single-trajectory models between one to eight trajectory groups were estimated for each of the six SOFA-domains. All outcome trajectories (i.e., SOFA-scores) up to third order polynomials terms (i.e., as a linear, quadratic and cubic function of time) were modelled, using the censored normal distribution. All possible combinations of polynomial order for each of the outcomes and for one to eight trajectory groups were evaluated.

Models were chosen based on the test of model adequacy as laid out by Nagin (3, 4); the average posterior probability for those assigned to a group based on the maximum posterior probability rule should exceed the 0.7 threshold of acceptability, the odds of correct classification should exceed 5, relative entropy value is recommended to be above the level of 0.8 indicating good separation between trajectory group, and group sizes should be no less than 5% of the total population.

We then compared the final single trajectory models of each of the six SOFA-scores. This indicated that that we needed between three (for the renal outcome), and up to five (for the CNS, respiratory and cardiovascular outcomes) trajectory groups to capture the different patterns of SOFA-domains over time.

The choice number of trajectories in the multi-trajectory model, inherently has a subjective component. To minimize this subjective assessment when choosing the number of trajectory groups, we fitted one to eight trajectories separately with multi-trajectory models and sought to minimize BIC, as described below. We started the multi-trajectory models with all terms defined as first order polynomials. We then used the parameter estimates as start values for the next model, and if model fit improved; iteratively added quadratic or cubic functions of time. All eight models were optimized, based on the model adequacy criteria described above. Comparing the eight fitted multi-trajectory models showed that BIC continued to decrease with increasing trajectory groups (see supplementary table 2 in this document) despite its property that punishes less parsimonious solutions. However, with more than five groups the decrease in BIC was relatively small. We were thus informed by BIC that only small changes occur with more than five trajectory groups, but BIC alone was not able to identify the optimal number of trajectory groups. This phenomenon is previously described by Nagin. (4) According to the recommendations, we graphed and visually examined all models and it showed that more than 5 trajectory groups did not add any distinct clinical features, rather trajectories were split up in smaller subsamples with little difference in organ failure patterns. For example, comparing models with 5 and 6 trajectory groups (Figure 5 and 6 in this supplement); indicated that trajectory groups 1-3 in the five-group model were divided in to 4 smaller groups in the six-group model. This did not, in our opinion add to the clinical understanding of organ failure after trauma. Models with more groups than seven all resulted in at least one group being smaller than the minimum size and were thus rejected. Graphs of the eight multi-trajectory models are presented in Figure 1-8 in this appendix. The choice was thus made between five to seven trajectory groups in the final model. As model parsimony and clinical interpretation is a recommended part of model selection, we concluded that the five-trajectory group model was the best balance between fit statistics such as BIC, the ability to capture the distinct organ failure patterns and group sizes above the minimum size.

Hence, a final multi-trajectory model with five different trajectory groups were chosen. The code for the final 5-group trajectory model is available from the authors on request.

*Model stability and global maximum*

To examine the stability and model robustness, we bootstrapped our entire sample of 660 patients with 200 iterations and reran the model each time. Further, there is always a risk that the maximum likelihood algorithm converges at a local, instead of a global maximum. In order to test this, we performed 200 iterations of the final multi-trajectory model with modified start values. We did not see any major differences in fit statistics or trajectory groups assignments after these analyses.

Finally, to evaluate model performance on unseen data, we used leave-one-out cross validation to calculate individual estimates over time by first training the model (estimating all model parameters) on the total cohort but excluding one individual, then using that individual’s data with the trained model parameters to estimate parameters, PPGM and trajectory group. Then we pooled the results of all individual holdout experiments to evaluate overall model performance. No major differences in model performance in terms of trajectory group size and individual patients’ group assignment were seen (data not shown).

| **Nr of trajectory groups** | **Best BIC** | **Best AIC** | **Average posterior probability (per trajectory group)** | **Percent of patients with posterior probability <70% (per trajectory group)** | **Odds of Correct Classification (per trajectory group)** | **Percent of sample size (per trajectory group)** | **Relative entropy** |
| --- | --- | --- | --- | --- | --- | --- | --- |
| 1 | 47365 | 47317 | 1 | 0 | - | 100 | - |
| 2 | 36884 | 36810 | .99/.99 | 0.7/ 0 | 212/ 381 | 62/ 38 | 0.987 |
| 3 | 34666 | 34550 | .99/.98/.99 | 0.3/ 3.5/ 0 | 360/ 55/ 136 | 46/ 22/ 33 | 0.983 |
| 4 | 33524 | 33359 | .99/ .98/ .99/ .98 | 0/ 3.5/ 1.8/ 1.2 | 489/ 47/ 83/ 57 | 45/ 22/ 8/ 25 | 0.980 |
| 5 (final model) | 32928 | 32720 | .99/ .99/ .99/ .98/ .96 | 1/ 0/ 2.2/ 1.0/ 7,5 | 145/ 179/ 85/ 53/ 25 | 45/ 13/ 20/ 15/ 6 | 0.982 |
| 6 | 32335 | 32088 | .99/ .98/ .98/ .99/ .97/ .98 | 1.7/ 3.2/ 4.2/ 0/ 4.0/ 2.6 | 69/ 46/ 39/ 577/ 38/ 64 | 36/ 14/ 18/ 12/ 15/ 6 | .974 |
| 7 | 32075 | 31785 | 98/ 97/ 99/ 99/ 93/ 93/ 98 | 1.2/ 5.1/ 1.1/ 0/ 9.9/ 7.8/ 3.2 | 72/ 33/ 68/ 174/ 15/ 13/ 54 | 36/ 18/ 14/ 8/ 12/ 8/ 5 | 0.965 |
| 8 | 31829 | 31499 | 98/ 98/ 98/ 98/ 91/ 98/ 95/ 98 | 1.7/ 2.6/ 2.6/ 2.1/ 16/ 0/ 8.3/ 3.1 | 40/ 52/ 51/ 47/ 10/ 84/ 19/ 59 | 17/ 36/ 11/ 7/ 6/ 7/ 11/ 5 | 0.966 |

Supplemental table 2. Fit statistics for different number of trajectory groups. BIC, Bayesian Information Criterion. AIC, Akaike Information Criterion.

*Supplemental figure 1. One trajectory group.*


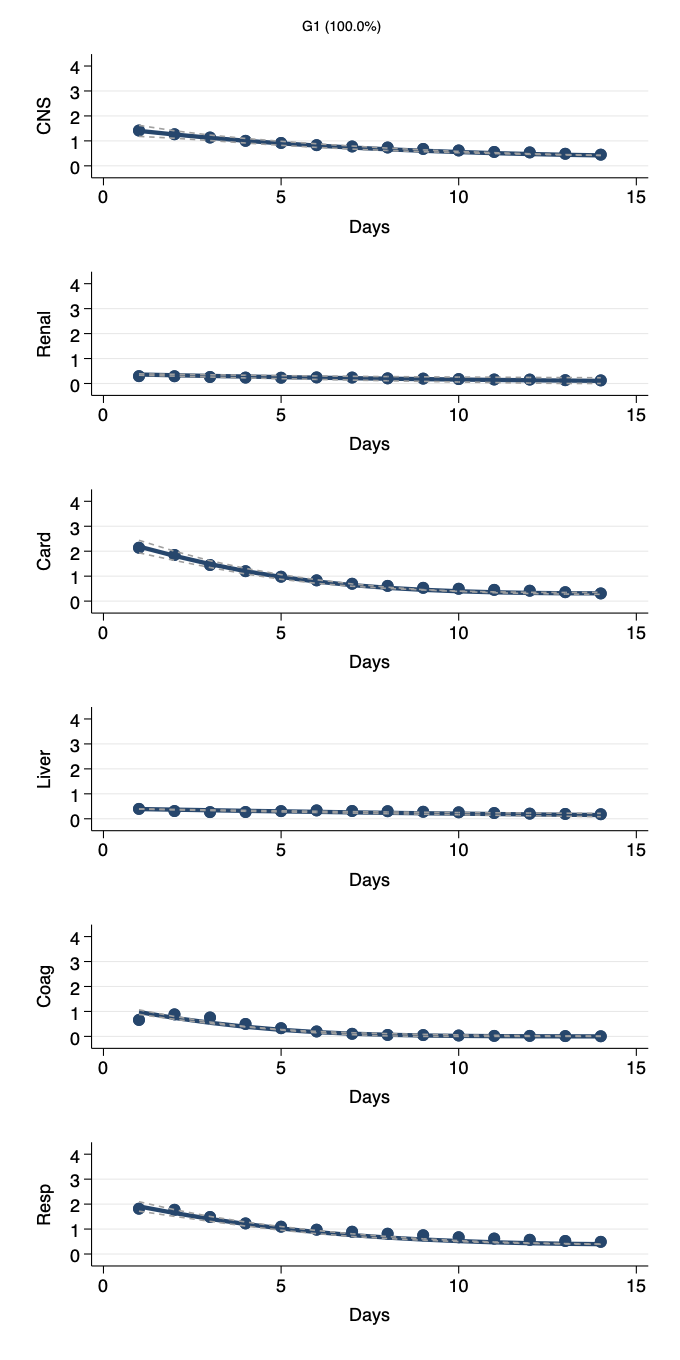


*Supplemental figure 2. Two trajectory groups.*


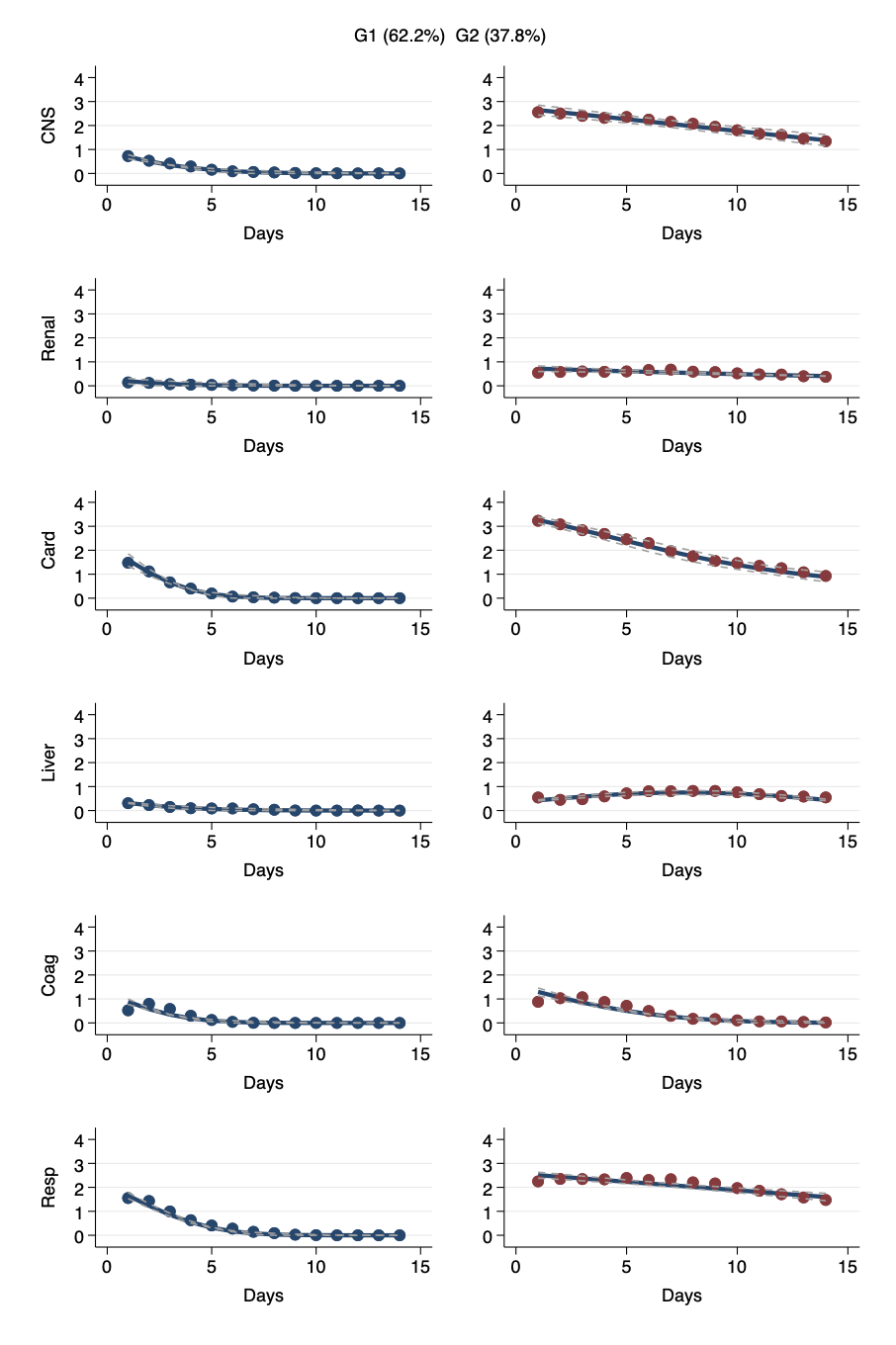


*Supplemental figure 3. Three trajectory groups.*


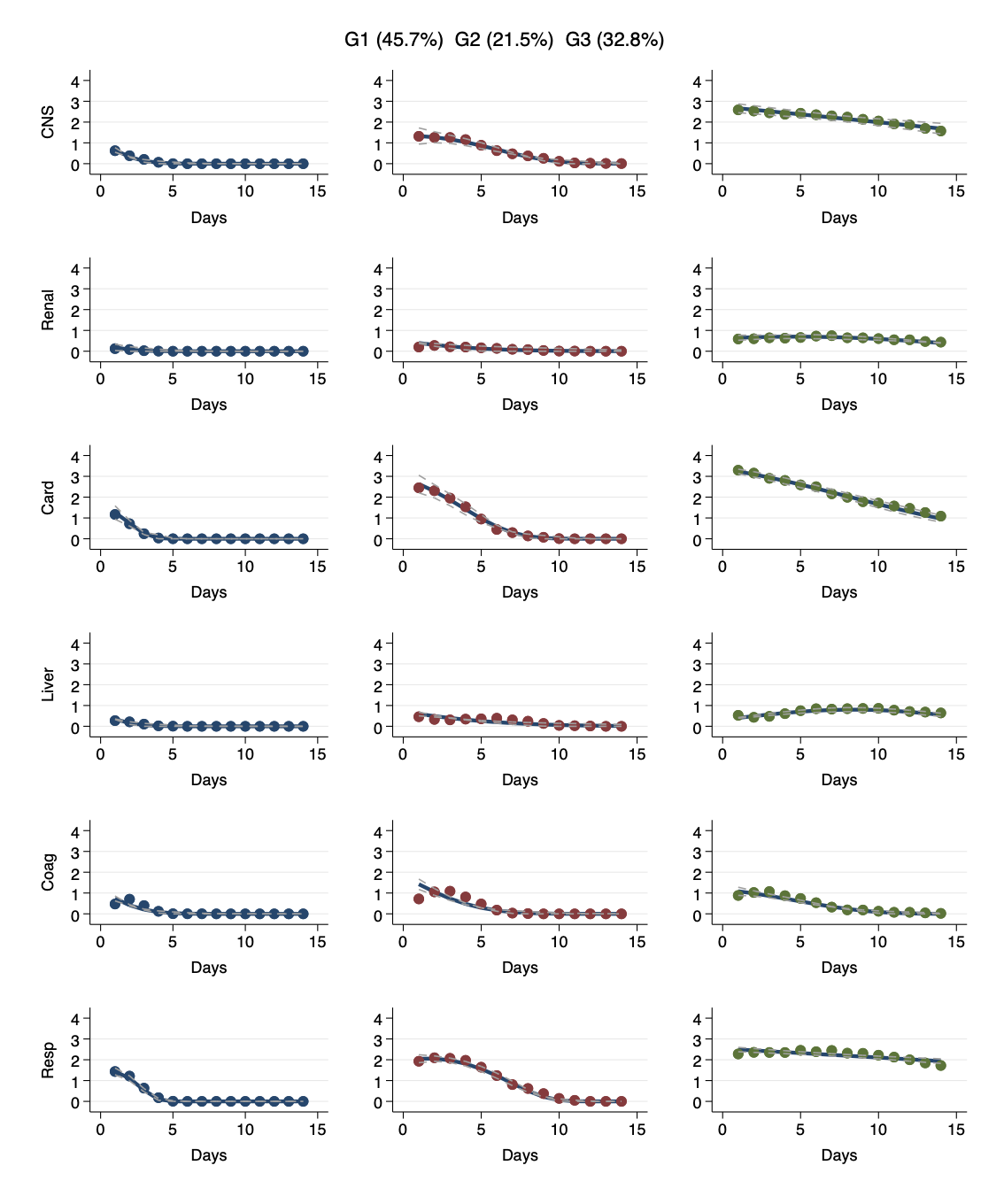


*Supplemental figure 4. Four trajectory groups.*


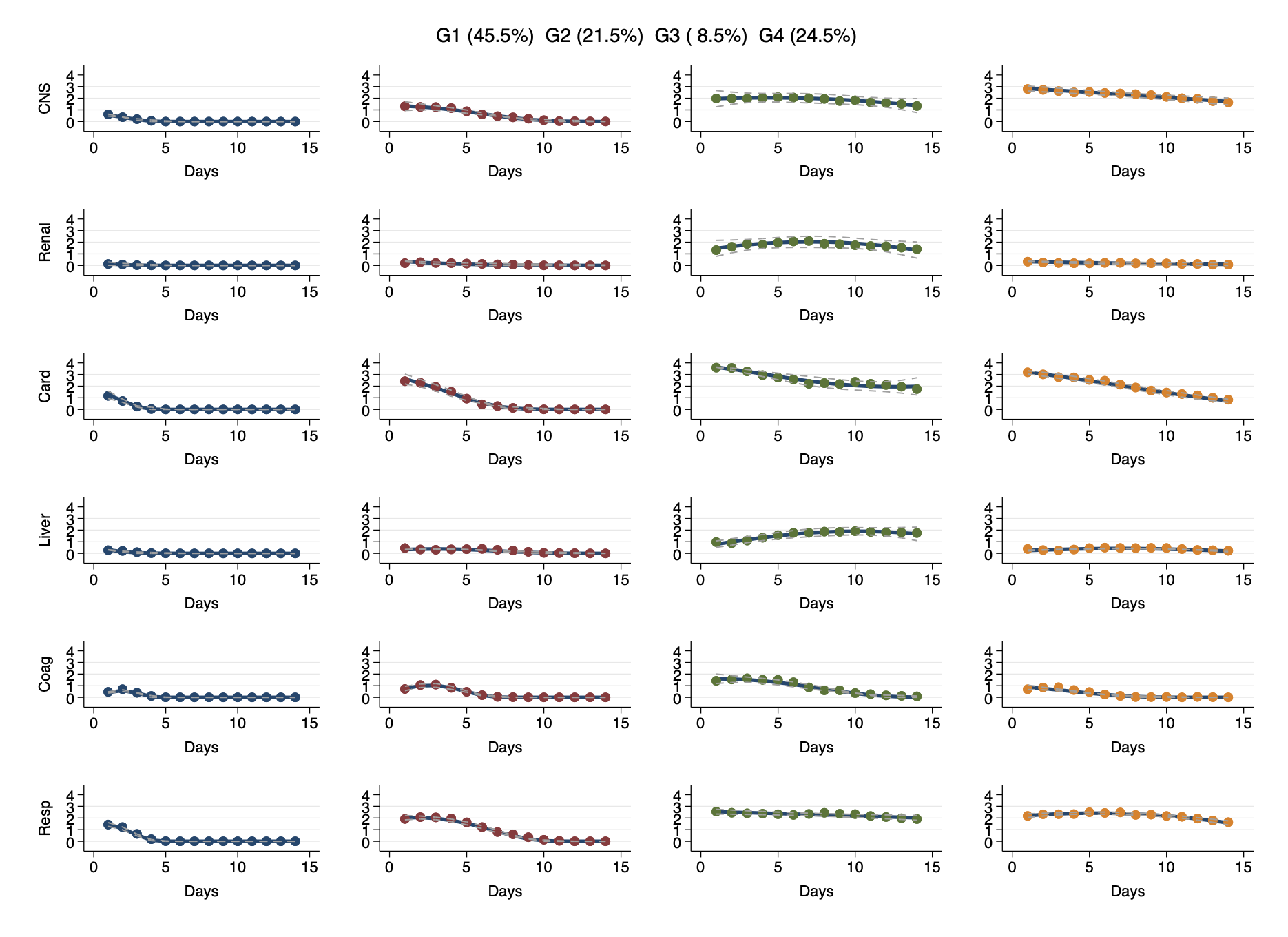


*Supplemental figure 5. Five trajectory groups (final multi-trajectory model, added for comparison).*


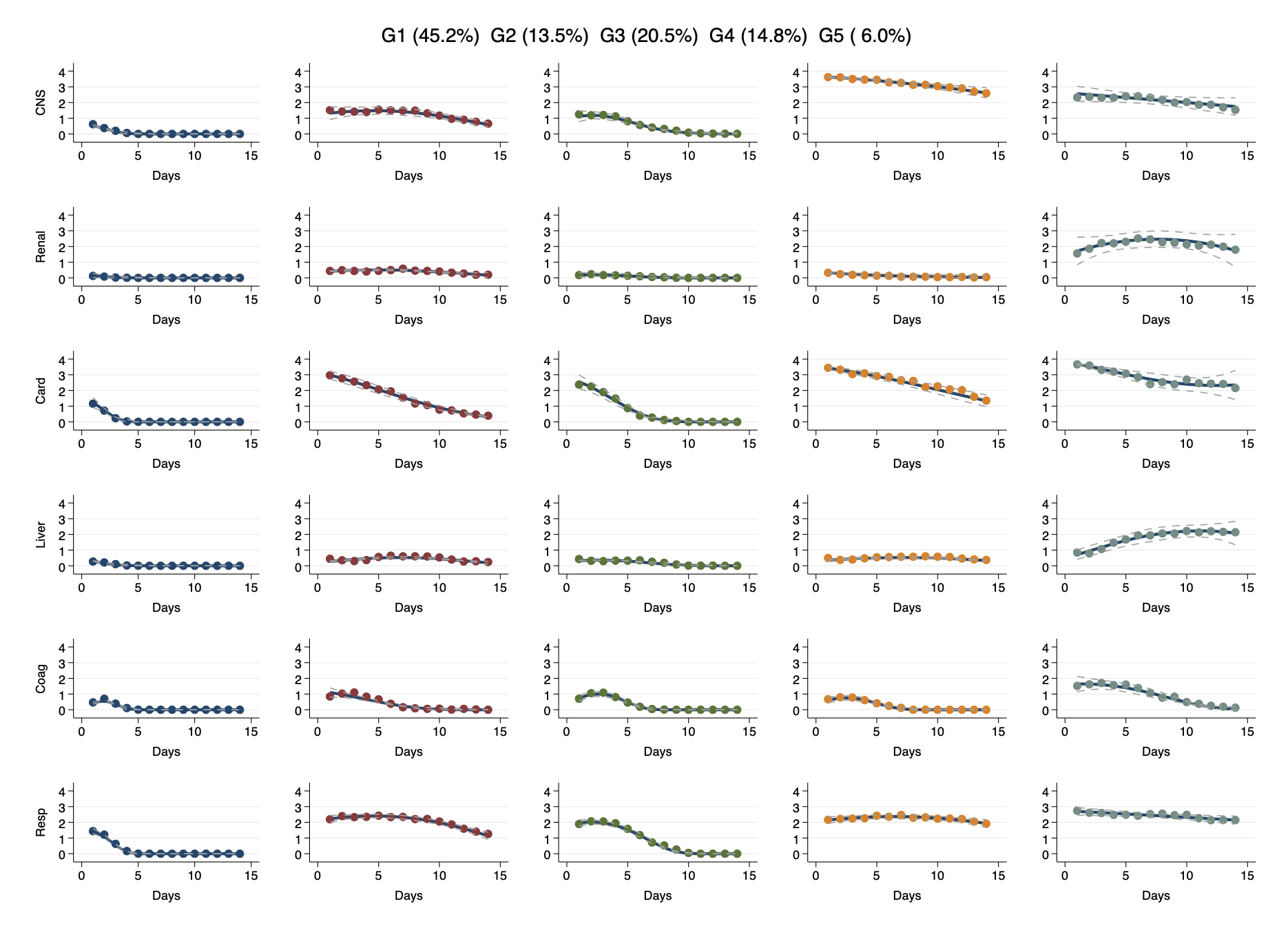


*Supplemental figure 6. Six trajectory groups.*

*
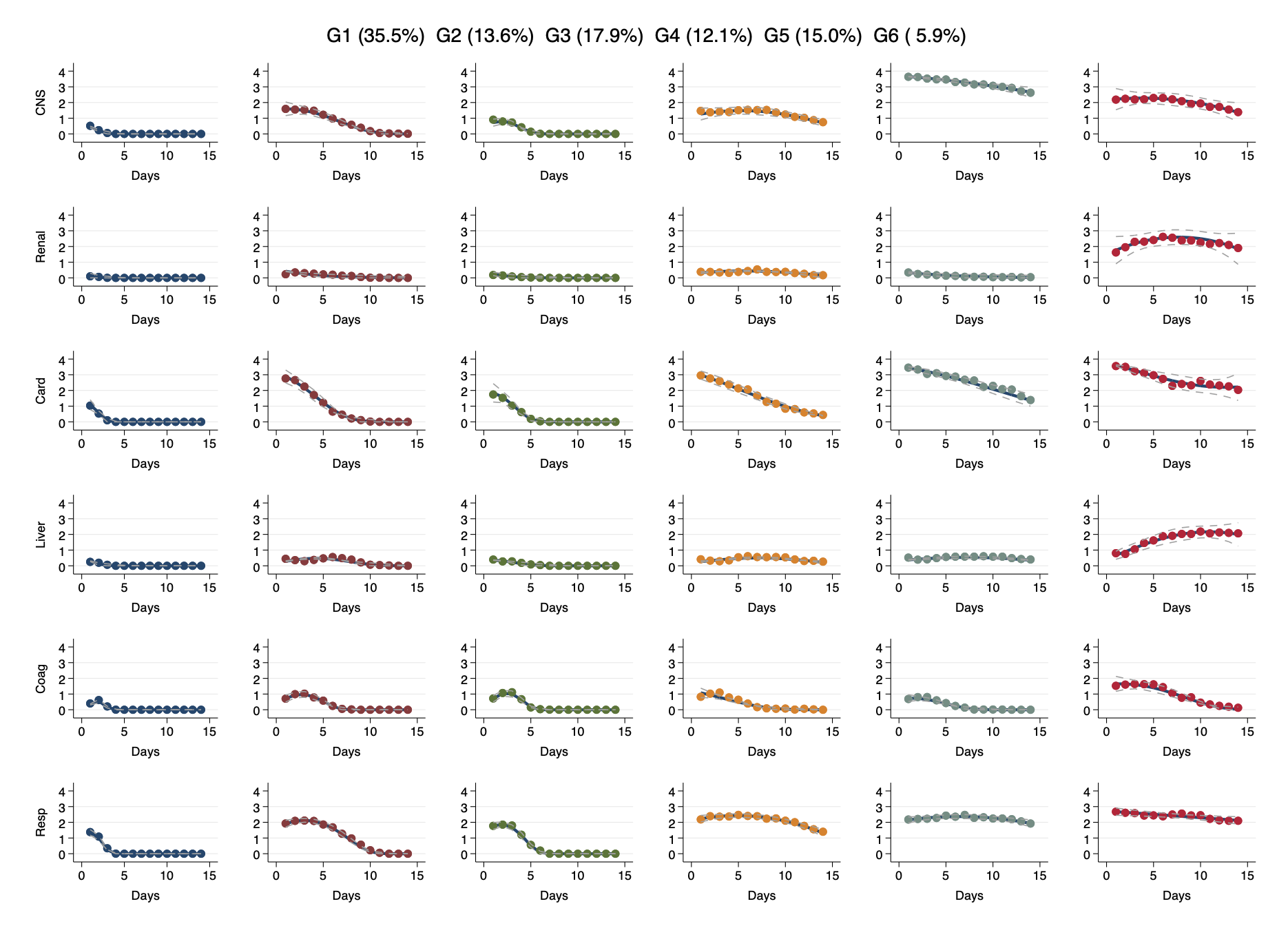
*

*Supplemental figure 7. Seven trajectory groups.*

*
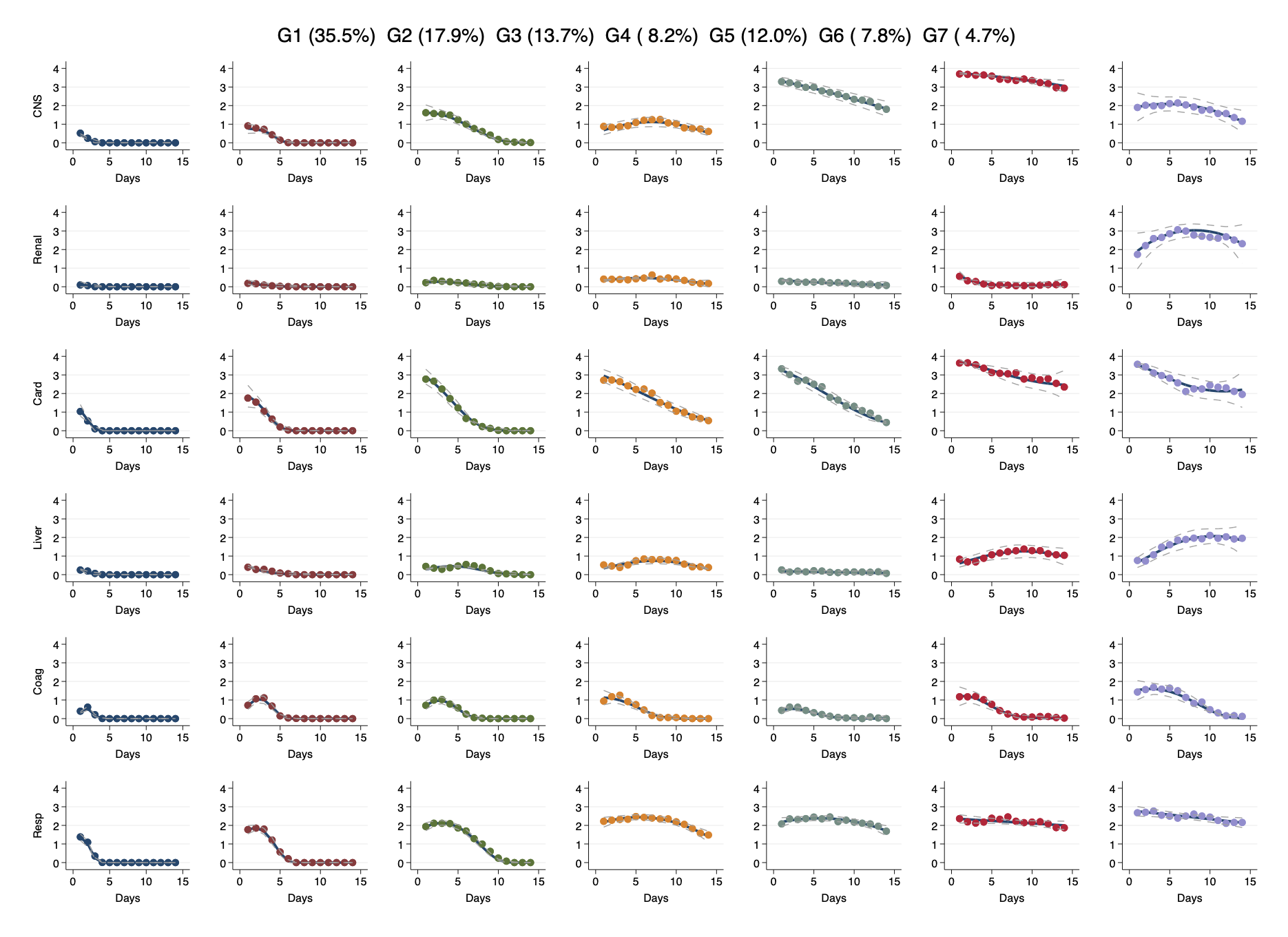
*

*Supplemental figure 8. Eight trajectory groups.*


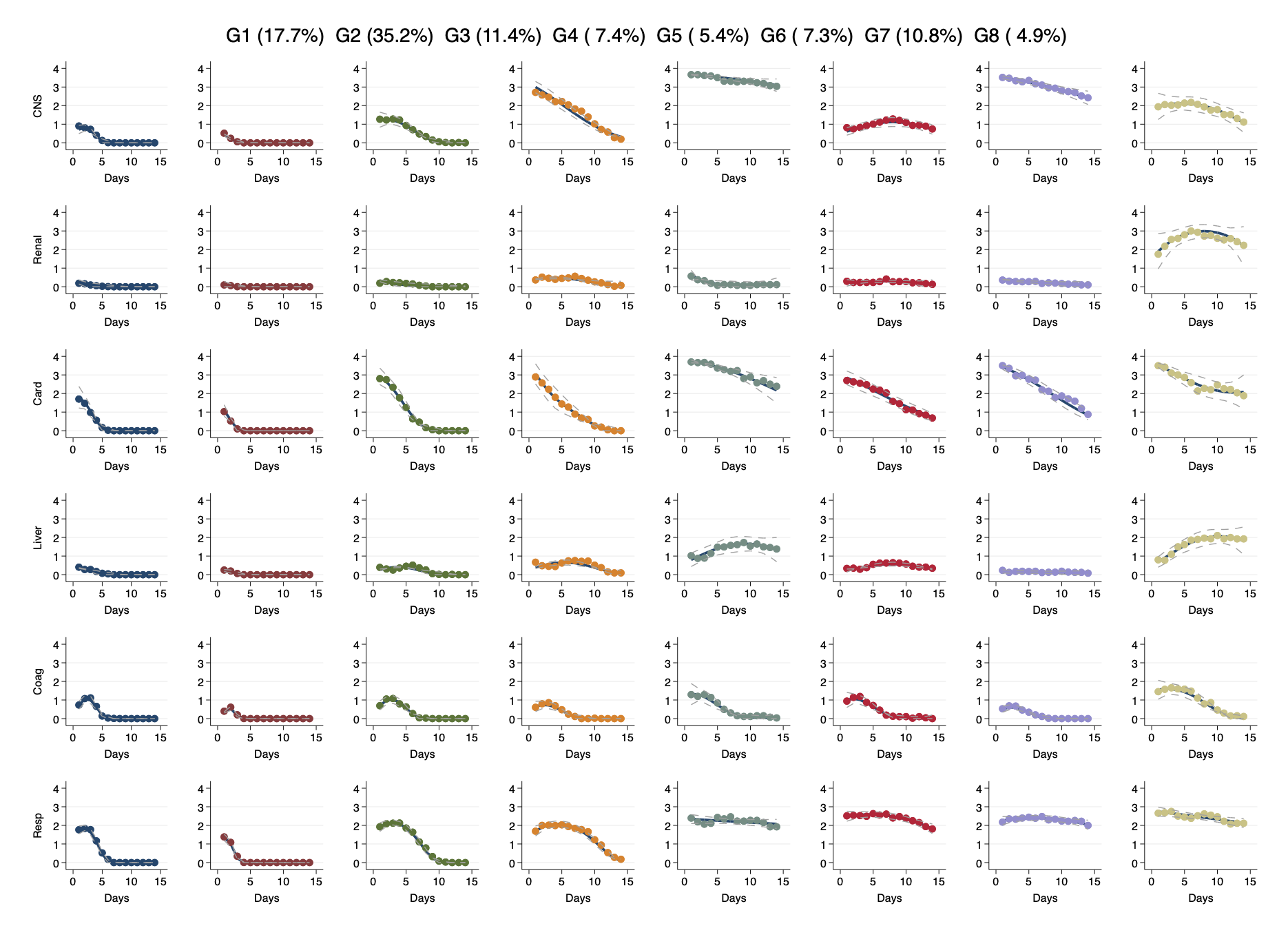


| GRoLTS checklist items |  |
| --- | --- |
| 1. Is the metric of time used in the statistical model reported? | Yes. SOFA scores are coded daily from trauma until day 14. |
| 2. Is information presented about the mean and variance of time within a wave? | Yes. SOFA scores were set daily at the same time for every time interval and trajectory group. Means of scores by day are reported for each trajectory group. |
| 3a. Is the missing data mechanism reported? | The mechanism of eligible but not included patients is in part due to nurse resources. These not included patients may not be completely at random as holidays and summers will find less included patients and trauma profiles may differ. Thus the cohort itself will be selected. For the final cohort; missing data were low and not and exclusion criteria. The few instances where there were missing data, we implemented multiple imputation using chained equations. Missing data in the selected cohort is expected to be at random. Missingness was not related to outcome. Missing data are reported for the full model.  A sensitivity analysis with full information maximum likelihood (i.e. no imputation was performed) was conducted with no major changes in trajectory group assignment or number of cases in each group. |
| 3b. Is a description provided of what variables are related to attrition/missing data? |  |
| 3c. Is a description provided of how missing data in the analyses were dealt with? |  |
| 4. Is information about the distribution of the observed variables included? |  |
| 5. Is the software mentioned? | Yes, we used TRAJ package in Stata v16.1 to fit 1-8 trajectory groups. Data were modelled assuming censored normal distribution. We modelled all possible combinations up to third order polynomials for 1-8 trajectory groups and compared the results. We did not consider covariates in specification of the models and further no multinomial regression was performed after class allocation. We reiterated the final model solution with altered start values to minimize the risk of converging to local maxima. To ensure that the groups are consistent we bootstrapped the sample 200 times, we did not see any major differences in fit statistics or trajectory groups assignments. |
| 6a. Are alternative specifications of within-class heterogeneity considered (e.g., LGCA vs. LGMM) and clearly documented? If not, was sufficient justification provided as to eliminate certain specifications from consideration? |  |
| 6b. Are alternative specifications of the between-class differences in variance–covariance matrix structure considered and clearly documented? If not, was sufficient justification provided as to eliminate certain specifications from consideration? |  |
| 7. Are alternative shape/functional forms of the trajectories described? |  |
| 8. If covariates have been used, can analyses still be replicated? |  |
| 9. Is information reported about the number of random start values and final iterations included? |  |
| 10. Are the model comparison (and selection) tools described from a statistical perspective? | We used several indices, including BIC, as well as substantive clinical interpretation when choosing the final model solution. See further explanation in Methods supplement. |
| 11. Are the total number of fitted models reported, including a one-class solution? | Yes, we show models fitted to 1 to 8 trajectory groups. |
| 12. Are the number of cases per class reported for each model (absolute sample size, or proportion)? | Yes. |
| 13. If classification of cases in a trajectory is the goal, is entropy reported? | Yes. |
| 14a. Is a plot included with the estimated mean trajectories of the final solution? | Yes. In figure 2. |
| 14b. Are plots included with the estimated mean trajectories for each model? | Yes, we have provided estimated mean trajectories for models with 1-4 and 6-8 trajectory groups in Methods supplement. |
| 14c. Is a plot included of the combination of estimated means of the final model and the observed individual trajectories split out for each latent reported? | No. This resulted in a incomprehensible graph. If needed, it can be supplied by the authors. |
| 15. Are characteristics of the final class solution numerically described (i.e., means, SD/SE, n, CI, etc.)? | We show class solution by means in figure 2. |
| 16. Are the syntax files available (either in the appendix, supplementary materials, or from the authors)? | We describe the operational procedure of the multitrajectory modelling in our methods section and in the Methods supplement. The syntax file for the final model is supplied by the authors on reasonable request. |

**References**

1. Elmer J, Jones BL, Nagin DS. Comparison of parametric and nonparametric methods for outcome prediction using longitudinal data after cardiac arrest. Resuscitation. 2020;148:152-60.

2. Nagin DS, Odgers CL. Group-based trajectory modeling in clinical research. Annual review of clinical psychology. 2010;6:109-38.

3. Nagin DS, Jones BL, Passos VL, Tremblay RE. Group-based multi-trajectory modeling. Stat Methods Med Res. 2018;27(7):2015-23.

4. Nagin D. Group based modeling of development. 2005.
